# Exploiting Pull-In/Pull-Out Hysteresis in Electrostatic MEMS Sensor Networks to Realize a Novel Sensing Continuous-Time Recurrent Neural Network

**DOI:** 10.3390/mi12030268

**Published:** 2021-03-05

**Authors:** Mohammad H Hasan, Amin Abbasalipour, Hamed Nikfarjam, Siavash Pourkamali, Muhammad Emad-Ud-Din, Roozbeh Jafari, Fadi Alsaleem

**Affiliations:** 1 Department of Earth and Space Sciences, Columbus State University, Columbus, GA 31909, USA; hhasan_mohammad@columbusstate.edu; 2 Department of Electrical and Computer Engineering, University of Texas at Dallas, Dallas, TX 75080, USA; axa147730@utdallas.edu (A.A.); hamed.nikfarjam@utdallas.edu (H.N.); Siavash.Pourkamali@utdallas.edu (S.P.); 3 Department of Computer Science and Engineering, Texas A&M University, College Station, TX 77843, USA; emaad22@tamu.edu (M.E.-U.-D.); rjafari@tamu.edu (R.J.); 4 Department of Biomedical Engineering, Texas A&M University, College Station, TX 77843, USA; 5 Department of Electrical and Computer Engineering, Texas A&M University, College Station, TX 77843, USA; 6 Durham School of Architectural Engineering and Construction, University of Nebraska—Lincoln, Omaha, NE 68182, USA

**Keywords:** neuromorphic computing, MEMS, Sensor Network, CTRNN

## Abstract

The goal of this paper is to provide a novel computing approach that can be used to reduce the power consumption, size, and cost of wearable electronics. To achieve this goal, the use of microelectromechanical systems (MEMS) sensors for simultaneous sensing and computing is introduced. Specifically, by enabling sensing and computing locally at the MEMS sensor node and utilizing the usually unwanted pull in/out hysteresis, we may eliminate the need for cloud computing and reduce the use of analog-to-digital converters, sampling circuits, and digital processors. As a proof of concept, we show that a simulation model of a network of three commercially available MEMS accelerometers can classify a train of square and triangular acceleration signals inherently using pull-in and release hysteresis. Furthermore, we develop and fabricate a network with finger arrays of parallel plate actuators to facilitate coupling between MEMS devices in the network using actuating assemblies and biasing assemblies, thus bypassing the previously reported coupling challenge in MEMS neural networks.

## 1. Introduction

Wearable devices promise great improvement in human quality of life by enabling health monitoring and diagnostics via human activity recognition (HAR), which is essential for fitness tracking, productivity assessment, and comfort management. Wearable devices rely on biological data measured through sensors such as accelerometers. The data points are then processed through complex machine learning schemes to determine the biological state. However, as wearable electronics are limited in power and space, complex machine learning approaches cannot be efficiently implemented locally. Instead, biological data are typically sent to the cloud for processing, causing power loss through wireless communication, and posing security risks in such systems.

Neuromorphic computing, first introduced by Mead [1], is a viable solution to the challenge of local computing in wearable devices. Neuromorphic computing started as an idea of using transistors in the subthreshold regime to simulate the response of biological neurons. More recently, this has evolved into a set of computing schemes that utilize analog devices to perform computing [2]. Neuromorphic computing has shown great power-saving potential while maintaining substantial computational ability [2]. Spiking neural networks (SNNs) are considered the most well-known neuromorphic computing schemes. In such a scheme a network of analog devices is used to produce a spiking response, like that observed in biological neurons [3]. Neuromorphic sensors have also been introduced previously to simulate the behavior of sensory organs and provide visual sensing [4], audio sensing [5], and olfactory sensing [6]. Such devices produce asynchronous spiking outputs corresponding to changes in the measured signals. However, for both neuromorphic implementations (sensors and SNNs) additional components are needed to translate the spiking signals into digital signals for further processing, increasing the footprint of such devices. Moreover, neuromorphic sensors require neuromorphic, spike-based processors, adding to the system size and power requirements [3].

**Figure 1:**
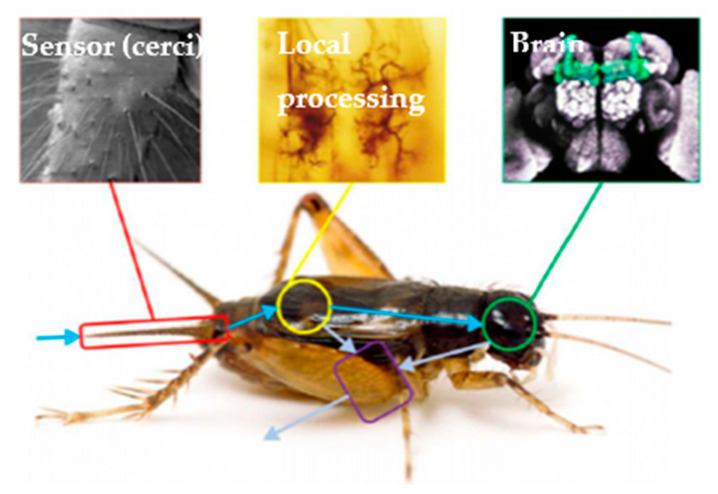
New findings in insect neural systems reveal that they have local integrated sensing and computing neurons to reduce computing demands at the central processing unit (the brain) [7].

Inspired by the neural system of very tiny biological systems, such as some insects as shown in Figure 1 [7] an alternative means of computing that addresses such concerns is colocalized sensing and computing. In this approach, some of the measured signals are preprocessed at the sensor level. Sensory information is consequently produced or post-processed by a digital processor. Microelectromechanical Systems (MEMS) sensors have been previously considered for this type of computing process. Networks of MEMS oscillators were shown to be able to perform computing through oscillator synchronization [8,9]. However, phase comparison between MEMS oscillators and the need to maintain specific initial conditions of oscillators are challenges that require addressing. More recently, a single MEMS device has been shown to perform computing through reservoir computing, by utilizing time-multiplexing to create temporally coupled virtual nodes [10,11]. However, in this approach, the response of the MEMS device is required to be sampled at very high rates (tens or hundreds of kHz). Furthermore, delayed feedback is required, which further complicates the required electronics.

In our previous work, we presented the novel use of MEMS electrostatic sensor dynamics with special geometric nonlinearities to naturally solve the continuous-time recurrent neural network (CTRNN) equations [12,13]. In that implementation, there is no need for a digital computer to solve the CTRNN equations. As an application, it was shown that the dynamics of eight coupled MEMS devices can be trained to perform a classification and object tracking of a mobile robot application [13]. In this paper, the concept of MEMS-based CTRNN is expanded to enable the use of almost any type of electrostatic MEMS to perform CTRNN computing. This new implementation relies on the hysteresis due to the Pull-in/Pull-out behavior that inherently exists in almost any parallel-plate electrostatic MEMS transducer. Furthermore, compared to previous works, the concept of colocalized sensing and computing eliminates the need for additional sensors at the network input layer and the need for capacitive measurement elements, thus reducing the network footprint.

The organization of this article is as follows: In Section 2 The theory for CTRNN and the use of MEMS Pull-in/out instability to perform CTRNN computation is introduced. In Section 3, we demonstrate through simulation model for a small network of off-the-shelf MEMS accelerometer sensors to perform a simple classification problem and we present the challenge for experimental implantation. In Section 4, we design and fabricate the first MEMS CTRNN to perform simultaneous sensing and computing. We also provide some preliminary results and motivate the need for more thorough parameter optimization for this novel network to perform classification problems experimentally. Finally, we provide conclusions and future work in Section 5.

## 2. Theory and Methodology

### 2.1. RNN vs. CTRNN

Recurrent neural networks (RNN), unlike traditional feed-forward neural networks (FFNN), utilize internal memory through self-feedback to preserve the sequences of input data during training [14,15]. Thus, the RNNs have shown great success in sensory applications such as image, video, and audio processing, as well as in optimization, associative memories, and controls [14]. A special, yet very complex form of RNN known as a CTRNN [16], uses differential equations to describe the activation level of the neurons (see Equation (1) below). To perform a certain classification problem, the self-coupling and cross-coupling weights between different neurons of a CTRNN are determined through the training performed during the design phase of the network.

(1) $$\dot{y_{i}}=f_{i}\left(y_{1},\ldots ,y_{N}\right)=\frac{1}{\tau_{i}}\left(-y_{i}+\sum_{j=1}^{N}w_{ij}\sigma \left(y_{j}\right)+h_{i}+I_{i}\right),i=1,2,\ldots ,N$$

where σ is an activation function, $\tau_{i}$ and *y_i_* are the time constant and activation level of neuron *i*, respectively, *w_ij_* is the connection strength between the *i*th neuron and the *j*th neuron, h is a bias term, *I_i_* is the input to the *i*th neuron, and the dot operator represents the time derivative.

**Figure 2:**
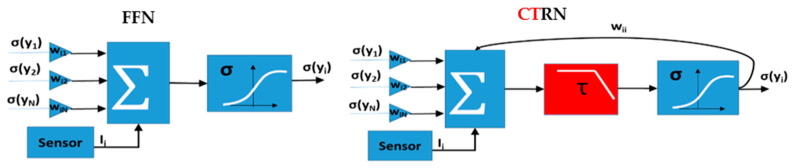
The differences between FFN and CT/RN. While CTRN and RN have internal memory through self-feedback, a CTRN approximates the response of a group of RNs by having a first-order differential equation.

Figure 2 shows schematic diagrams comparing the structure of a single feedforward neuron (FFN), a recurrent neuron (RN), and a continuous-time recurrent neuron (CTRN). The schematics show that while having self-feedback is the main difference between CT/RN and the FFN, the differential equation is the main difference between the RN and the CTRN. The first-order differential equation with a time constant *τ* of the CTRN model acts as a low-pass filter. The function of $\tau_{i}$ is to produce a resistance to reject the input from other neurons and try to maintain the influence of previous inputs on the neuron. Larger $\tau_{i}$ means stronger resistance and a slower activation process. In other words, a neuron with a large time constant attempts to store the history information and needs a longer time to accept new inputs. The value of $\tau_{i}$ thus has a profound impact on the overall model learned by the CTRNN network. Moreover, it provides the CTRNN a learning capability comparable to the state-of-art advanced, yet complex, recurrent neural networks such as Long Short-Term Memory (LSTM) neural network. As such, CTRNNs have emerged as a very attractive machine learning option as they require fewer neurons for high-level learning. For example, a CTRNN made of only four CTRNs was needed to learn eight wrist trajectories from its acceleration measurements [17], where 128 RNs were needed to perform a similar task [18]. However, CTRNNs are computationally expensive for real-time implementation as they require simultaneous solutions of highly coupled multiple differential equations. This makes them unsuitable for many emerging applications such as wearable devices with limited memory and processing capabilities [19].

### 2.2. MEMS-Based CTRNN Approach

**Figure 3:**
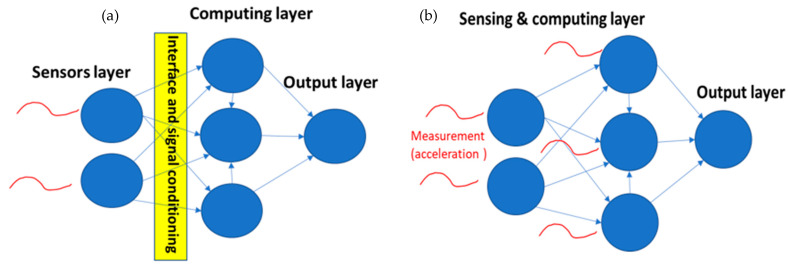
(**a**) Our previous attempt for building a MEMS CTRNN follows a typical machine learning approach that separates sensing and computing. While it provides an efficient way to do computing, it still requires the complex sensor reading and interface between the input and output layers. (**b**) This paper’s contribution is to investigate and demonstrate a MEMS CTRNN that no longer separates between the input and output layers.

To overcome the challenges, in previous work, we have identified nonlinearity and hysteresis as essential properties for CTRNNs to perform computing. Thus, we have shown that systems exhibiting these properties, such as a network of coupled bi-stable MEMS devices, are candidates for performing CTRNN computing in an analog fashion [12,13]. However, while that work demonstrated an efficient way to perform CTRNN computing using MEMS devices, it followed a typical machine learning structure that separates the input (sensor) layer from the computing layer (Figure 3a). As MEMS devices were originally designed to be sensors, in this work, we expand the MEMS novel computing concept to allow a MEMS bi-stable network to perform simultaneous sensing and computing (Figure 3b). Thus, eliminating the need for the complex sensor interfaces and signal conditioning circuits to perform similar computation.

### 2.3. Modeling

**Figure 4:**
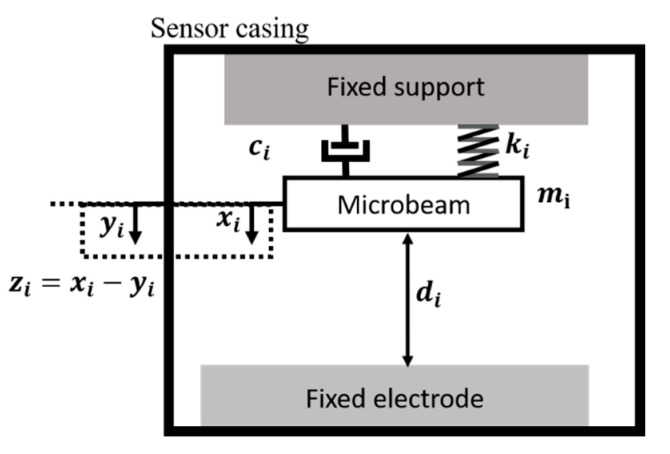
MEMS Schematic.

In the new MEMS sensing and computing implementation, we approximate the response of each MEMS device in the N-MEMS network as a single-degree of freedom spring-mass-damper system, shown in Figure 4 and governed by (1):

(2) $$m_{i}{\ddot{z}}_{i}\left(t\right)+c_{i}z_{i}\left(t\right)+k_{i}z_{i}\left(t\right)=\frac{\varepsilon A{\left(\sum_{j=1}^{N}w_{ij}^{*}V_{j}U\left(z_{j}\left(t\right)-d_{j}\right)+V_{bi}\right)}^{2}}{2{\left(d_{i}-z_{i}\right)}^{2}}-m_{i}\ddot{y}\left(t\right)$$

where $z_{i}\left(t\right)=x_{i}\left(t\right)-y_{i}\left(t\right)$ is the relative deflection of the *i*th MEMS device at time t, computed as the difference between the absolute MEMS deflection $x_{i}\left(t\right)$ and the base (sensor casing) displacement $y_{i}\left(t\right)$. The ith MEMS device in the network has a mass $m_{i}$, damping constant $c_{i}$ and stiffness $k_{i}$. The surface area of each MEMS electrode is $A_{i}$ and the separation between the stationary and moving electrodes of the MEMS devices, when at rest, is $d_{i}$. Each MEMS device is electrostatically actuated using a signal $V_{i}=\sum \left(w_{ij}V_{j}U\left(z_{j}\left(t\right)-d_{j}\right)\right)+V_{bi}$ composed of a bias voltage $V_{bi}$ and an external weighted signal from the other MEMS devices in the network $w_{ij}^{*}V_{j}U\left(x_{j}\left(t\right)-d_{j}\right)$, where $U\left(x_{j}\left(t\right)-d_{j}\right)$ is a unit step function that activates when $x_{j}\left(t\right)\geq d_{j}$, representing a switching action and $w_{ij}^{*}$ is the connection weight from the *j*th MEMS device to the *i*th MEMS device. Each MEMS device in the network may experience pull-in instability if the total applied voltage (pull-in voltage) produces an electrostatic force that exceeds the mechanical stiffness of the structure. This leads to the collapse of the MEMS structure and the closing of an electrical circuit (ON state). However, due to internet hysteresis behavior, the voltage needs to be reduced to a value smaller than the pull-in voltage (release voltage) to release the proof mass (OFF state). To simulate the impact of pull in/out hysteresis in the MEMS network response in (2), we limit the MEMS deflection to a threshold value $x_{s}$, using mechanical stoppers, where $0.33d<x_{s}$ < d, where higher $x_{s}$ values indicate more hysteresis. In real MEMS implementation, hysteresis can be controlled by the thickness of the thin intermediate dielectric layer on the substrate [20,21].

To rewrite (2) in a similar form to the CTRNN equation in (1), we first non-dimensionalize (2) using the nondimensional parameters in (3):

(3) $$\hat{z_{i}}=\frac{z_{i}}{d_{i}},\hat{t}=\frac{t}{T_{i}},T_{i}=\frac{1}{\omega_{n_{i}}},\zeta_{i}=\frac{c_{i}}{2m_{i}\omega_{n_{i}}}$$

Applying the substitutions in (3) to (2) yields (4):

(4) $$\hat{\ddot{z_{i}}}+2\zeta_{i}{\hat{\dot{z}}}_{i}+{\hat{z}}_{i}=\frac{\varepsilon A{\left(\sum_{j=1}^{N}w_{ij}^{*}V_{j}U\left({\hat{z}}_{j}\left(t\right)-1\right)+V_{bi}\right)}^{2}}{2d_{i}^{3}k_{i}{\left(1-\hat{z_{i}}\right)}^{2}}-\frac{m_{i}}{\omega_{n}^{2}{}_{i}d_{i}}\ddot{y}\left(t\right)$$

where $\omega_{n}{}_{i}=\sqrt{k_{i}/m_{i}}$ is the natural resonance frequency of the ith MEMS device, and $\zeta_{i}$ is its damping ratio. One can show through dimensional analysis that, the first term in (4) can be dropped if the MEMS resonance frequency is sufficiently high, for a given damping ratio. This condition is easy to satisfy when the MEMS device is operated under atmospheric pressure due to the prevalence of squeeze-film damping [12]. Therefore, (4) can be rewritten in a form like the CTRNN equation, as follows:

(5) $$\tau_{i}{\hat{\dot{z}}}_{i}\left(t\right)=-{\hat{z}}_{i}\left(t\right)+\sigma_{M}\left(\sum_{j=1}^{N}w_{ij}V_{j}U\left({\hat{z}}_{j}\left(t\right)-1\right)+\theta_{i},{\hat{z}}_{i}\left(t\right)\right)+I_{i}\left(t\right)$$

where $\tau_{i}=2\zeta_{i}$ is the MEMS time constant, $I_{i}\left(t\right)=\frac{m_{i}}{\omega_{n}^{2}{}_{i}d_{i}}\ddot{y}\left(t\right)$ is the acceleration input to the MEMS device, $w_{ij}=\frac{\varepsilon Aw_{ij}^{*}}{d_{i}\sqrt{2k_{i}}}$ is the effective connection weight between the MEMS devices, $\theta_{i}=\frac{\varepsilon AV_{b_{i}}}{d_{i}\sqrt{2k_{i}}}$ is the bias signal and $\sigma_{M}\left(\alpha ,\beta \right)=\alpha^{2}/{\left(1-\beta \right)}^{2}$ is a nonlinear transformation corresponding to the nonlinear electrostatic forcing on the MEMS device. The parameters to be optimized in the MEMS CTRNN to achieve a certain functionality are $\omega_{n_{i}}$, $k_{i}$, $d_{i}$ $V_{b_{i}}$, and $w_{i,j}^{*}$.

### 2.4. Weight Implementation

To achieve coupling with adjustable weights between the MEMS devices in a computing network, we have used operational amplifiers.^13^ However, this approach requires extra electronics and power and scales poorly as the network size increases. Moreover, it cannot be used to represent negative weights as while an operational amplifier can invert the input voltage polarity, a MEMS according to (4) only responds to the square of the voltage. To address this challenge, we have adopted the electrostatic mechanical coupling mechanism. This approach has been already used by our team to realize a digital mechanical MEMS accelerometer [22].

**Figure 5:**
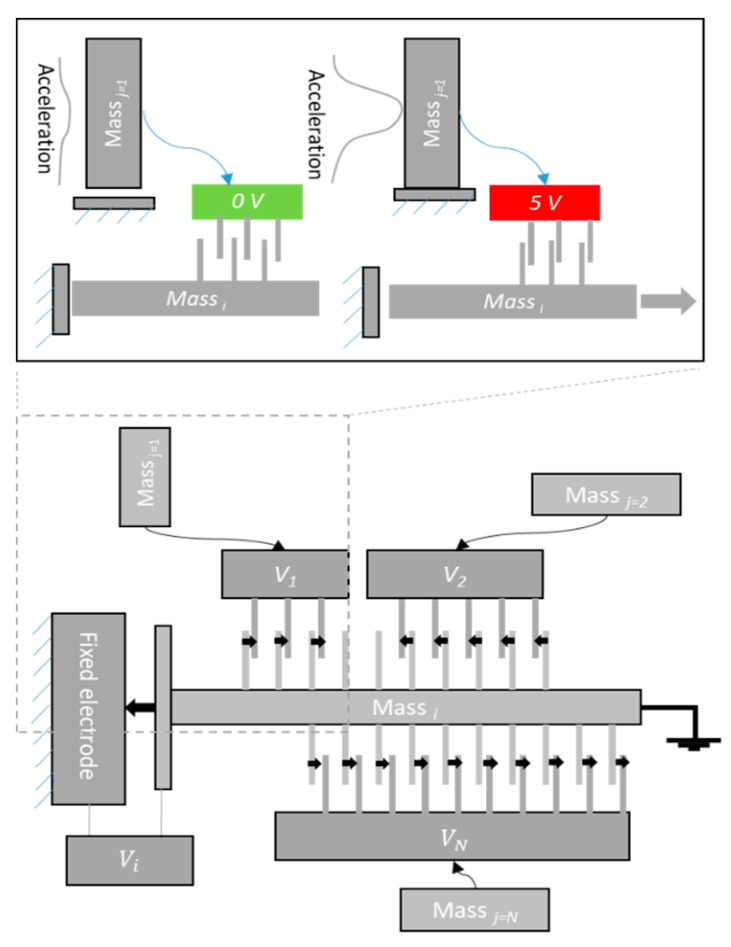
The finger arrays approach to realize electrostatic coupling between the MEMS CTRNs.

In this approach, electrostatic parallel plate finger arrays will be adopted to realize the coupling between the neurons of the MEMS-based CTRNN. For example, in the sensing and computing layer, a proof mass *i* is coupled to other proof masses by different sets of fingers as shown in Figure 5. The activation voltage of each *j* set of fingers acting on proof mass *i* is controlled by the corresponding proof mass *j* status. Thus, the total electrostatic forces acting on a proof mass *i* can be described by:

(6) $$F_{ic}=\frac{\varepsilon A_{1}\left(\sum_{j=1}^{N}\mp n_{j}V_{out,j}^{2}\right)}{2d_{1}^{2}}$$

where *V_out,j_(u_j_)* is the output voltage from the *j*th proof mass, and *n_j_*, *A_1_*, and *d_i_* are the number of parallel fingers controlled by the proof mass *j*, the overlapping area of the parallel fingers, and the nominal separation between the fingers, respectively.

The coupling effect may be positive or negative depending on the relative position between the stationary fingers and the moving fingers (attached to proof mass). For example, for the proof mass*_i_* shown in the figure, the voltage signal for fingers activated by proof mass*_j=2_* are associated with a positive effect because they produce a force that moves mass*_i_* toward its fixed electrode. On the other hand, the fingers activated by mass*_j=1_* are associated with a negative effect as it produces a force in the opposite direction. The former operation is demonstrated in the top schematic of Figure 5, where the mass*_j=1_* that is oriented along the y-direction may receive enough acceleration to bring it into contact with its fixed substrate. This in turn activates the applied voltage *V_1_* on its corresponding fingers acting on mass_i_ to pull it away from its fixed substrate.

## 3. Waveform Classification Using a Commercial Off-the-Shelf Accelerometer

**Figure 6:**
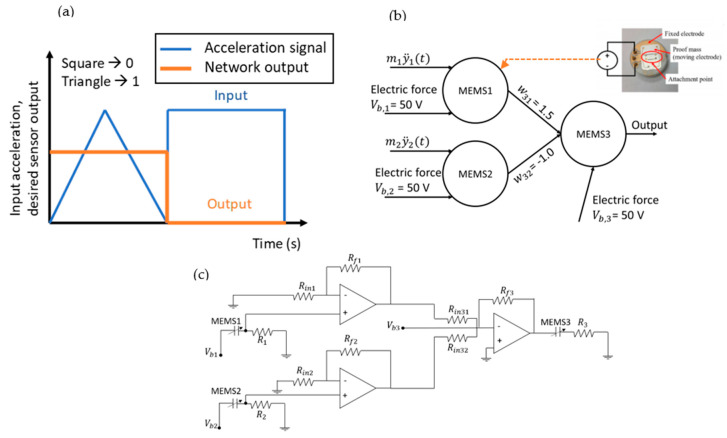
Classification task considered in this work. (**a**) Visualization of the binary classification problem. (**b**) MEMS network used for classification. The network is composed of three identical devices. Two devices receive an input acceler-ation signal and one device performs classification. (**c**) A connection circuit for the MEMS network.

Classification is one of the most popular tasks in the machine learning literature. For this work, we consider a simple classification task as a test for the computational potential of a network of MEMS devices. The task here involves the non-trivial problem in the literature [23,24] to classify an input waveform into either ‘Square’ signal or ‘Triangular’ signal, as shown in Figure 6. The input waveforms are supplied as acceleration waveforms. We note here that, unlike other physical implementations of neural networks where inputs are electrical signals, the MEMS network simultaneously performs sensing and computing. For the MEMS CTRNN to perform the computational task properly, the size of the network and the connection weights between the MEMS devices are optimized. Optimization was performed manually by starting from a ladder diagram optimization scheme, assuming each MEMS device is a relay switch with no memory. Under that assumption, five MEMS devices are required to perform the computational task. The number of MEMS devices required is reduced to three by taking advantage of the dynamics of MEMS devices, namely inertia and pull in/out hysteresis.

The bias voltages were chosen such that $V_{b,1}>V_{b,2}$ to force MEMS1 to pull-in ahead of MEMS2 when supplied by a ramped signal. MEMS1 and MEMS2 pull-in nearly simultaneously when a square acceleration signal is applied to the CTRNN. The connection weights between the MEMS devices in the network are also optimized manually by taking advantage of the ‘selection properties’ of a CTRNN [12,16]. Due to selection, the influence of input signals depends on the amplitude of the input signals as well as their temporal order. We note here that, due to our chosen method of weight optimization, the MEMS CTRNN will be able to classify any quasi-static acceleration signal. However, at acceleration frequencies close to the natural frequencies of MEMS1 and MEMS2, this method fails. Other optimization methods would be required to enable the classification of such signals.

For our task, a model for a network of identical commercial off-the-shelf accelerometer doubly cantilever MEMS accelerometer devices fabricated by Sensata technologies was used. The accelerometer is designed to measure low g acceleration, but if a high bias voltage is applied, it can pull-in and acts as a switch. The network is assumed to be coupled using operational amplifiers [12], in a fashion similar to that shown in Figure 6c. Here, the resistor values would be chosen for two purposes: reducing the current follow at pull-in; and tuning the connection weights between the MEMS devices. The parameters of the MEMS devices are presented in Table 1. This MEMS device is shown in the insert of Figure 6b. The MEMS devices in this circuit can be connected in series to large resistor to reduce the current following in the circuit at pull-in, which would otherwise burn the MEMS circuit. Additional information about the sensor and its model can be found in [12]. Here, it is assumed that MEMS1 and MEMS2 are input neurons, directly influenced by the acceleration signal. MEMS3, however, to simplify the calculation, is designed to be oblivious to the acceleration signal. This can be achieved by rotating MEMS3 such that the acceleration signal is perpendicular to the MEMS motion.

**Figure 7:**
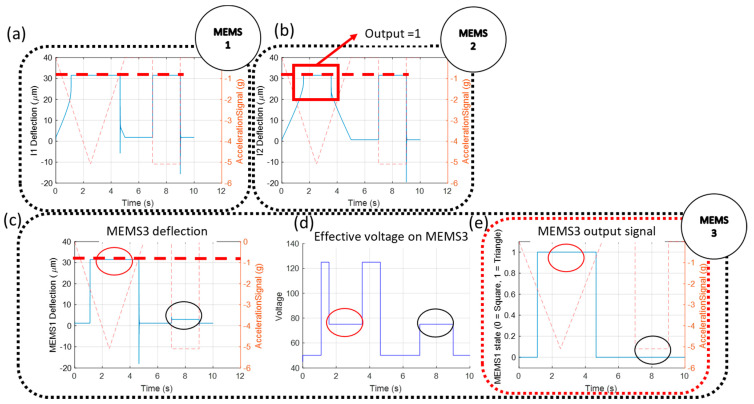
Classification test results showing the response of MEMS1 (**a**), MEMS2 (**b**) and MEMS3 (**c**). (**d**) The effective voltage acting on MEMS3 $V_{3}\left(t\right)$. (**e**) The state of MEMS3 when subject to a triangle or a square signal.

As a demonstration, the MEMS CTRNN is subjected to a sequence of a square and triangle signal with an amplitude $\ddot{y}=-5g$. The results of the MEMS CTRNN are shown in Figure 7. This figure is produced using a Matlab code, assuming that each MEMS device acts as a perfect switch with output $V_{out,i}=V_{i}U\left(x-x_{s}\right)$. The simulated shock signal excites both MEMS1 and MEMS2 (Figure 7a,b, respectively). Initially, when a triangle signal is observed, MEMS1 pulls-in (at around −2 g) first, ahead of MEMS2, due to its higher bias voltage. Consequently, MEMS3 pulls-in. When the acceleration signal ramps to −3 g, MEMS2 pull-in. Since MEMS2 has a negative connection weight, it reduces $V_{3}\left(t\right)$ to a value below the MEMS3 pull-in voltage. However, this reduction is insufficient to release MEMS3, due to the hysteresis at pull-out. Thus, MEMS3 remains pulled-in until the acceleration amplitude is reduced to below −2 g. Hence, despite MEMS1 and MEMS2 eventually pulling-in when they experience a triangle-shaped acceleration signal, the difference in pull-in timing ultimately results in triangle classification.

Alternatively, when a square signal is encountered, MEMS1 and MEMS2 experience a sudden and immediate change in amplitude, which results in them pulling-in (nearly) simultaneously. In this case, the voltage acting on MEMS3 is immediately equal to $w_{31}V_{b,1}+w_{3,2}V_{b,2}+V_{b,3}$ (noting that $w_{31}>0$, $w_{32}<0$). By design, this voltage is insufficient to pull-in MEMS3. Therefore, the output of MEMS3 remains low and square classification is performed. Interestingly, MEMS inertia is beneficial in this computing scheme as inertia prevents MEMS3 from pulling-in if MEMS1 is pulled in momentarily before MEMS2. Moreover, inertia allows this scheme to be performed to classify imperfect square signals, such as signals generated from a shaker, which tend to be trapezoidal, assuming the signal ramp is sufficiently steep since the MEMS devices will slightly lag the input signal.

The results from Figure 7 also clearly demonstrate the importance of hysteresis in a MEMS CTRNN as inputs of equal amplitudes may lead to significantly different behaviors depending on past information. (see the areas marked by the red circle and black dashed circle in Figure 7a–d, in which MEMS1 and MEMS2 are simultaneously pulled-in, yet MEMS3 can assume two different configurations).

It is worth mentioning that while the above simulated results provide great insight into the possibility of realizing a MEMS sensing and computing CTRNN and its working principle, a physical implementation of such a network using the commercial MEMS accelerometers network is not warranted. The commercial MEMS accelerometers are fabricated and packaged individually without any sort of mechanical coupling. Thus with this configuration it is hard to implement the negative weight (*w_32_* in Table 1). The limitation of the commercial MEMS accelerometers has motivated the need to fabricate a customized design that utilizes mechanical coupling to achieve negative weight.

## 4. Waveform Classification Using a Customized MEMS Network

**Figure 8:**
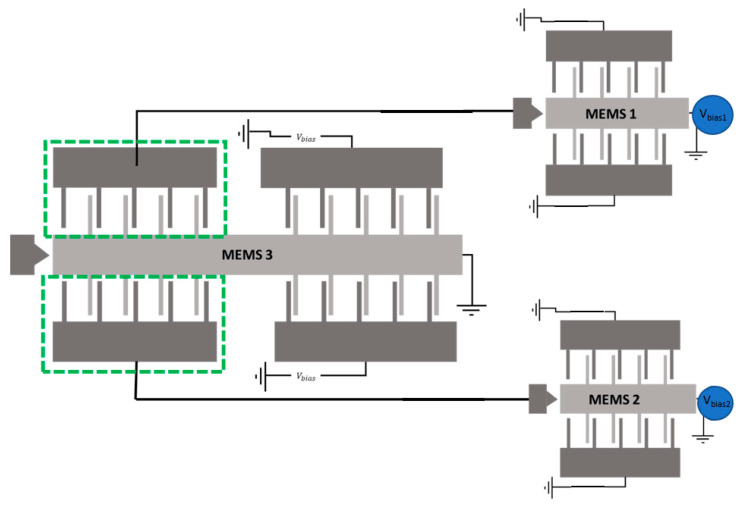
A schematic for the customized MEMS CTRNN to perform waveform classification. The schematic highlights the coupling through fingers between the three MEMS.

**Figure A1:**
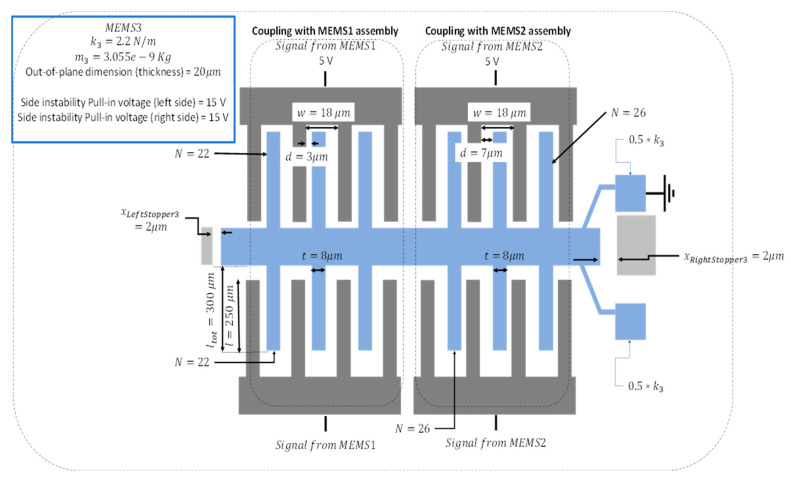
Design of the computing MMES device (labeled MEMS3 in Section 4), including information about the total stiffness of the MEMS device and the pull-in voltage.

**Figure A2:**
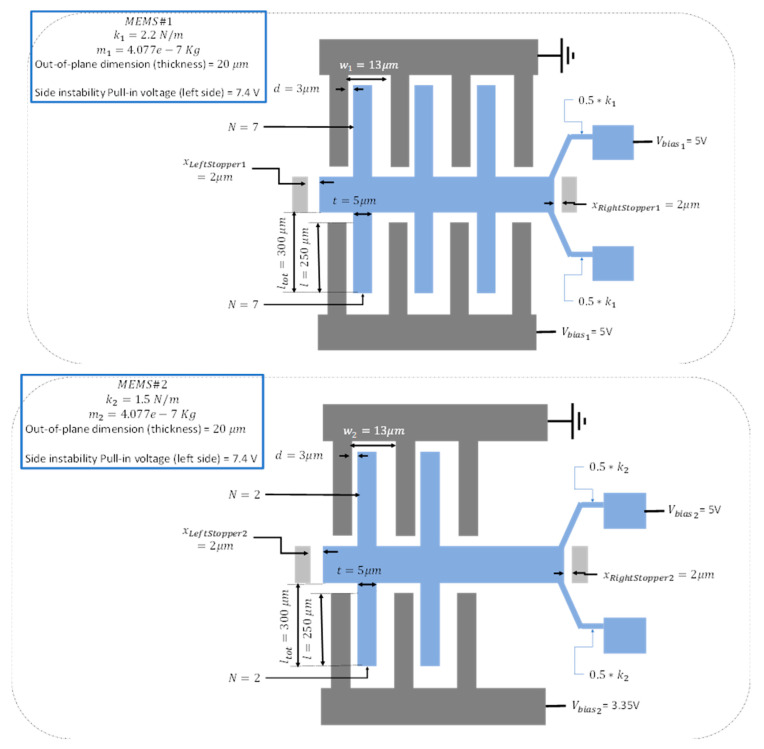
Design of the input layer MMES device (labeled MEMS1 and MEMS2 in Section 4), including information about the total stiffness of the MEMS devices and the pull-in voltages. Top figure: MEMS1, bottom figure: MEMS2.

In this section, a novel design of a MEMS network to perform sensing and computing is presented. A schematic for the full network is presented in Figure 8 and a detailed schematic with dimensions of each MEMS in the network is in Figure A1 and Figure A2 in the Appendix A and Appendix B. Figure 8 shows the network is made of two input MEMS sensors, each biased with a different voltage, to enable a different response to the applied acceleration. If the applied acceleration exceeds a threshold value, the MEMS will act as an ON switch that will activate a set of fingers on the computing MEMS (MEMS3). The bias voltages for each MEMS device and the number of fingers were manually tuned so that the MEMS3 will be pulled in (ON switch) when the applied acceleration is a triangular signal. Otherwise, it will be off. We note here that the output terminals have been designed at the proof mass contact point (represented by the triangular edges in Figure 8) to reduce the contact gap and minimize the risk of stiction. These contact tips additionally serve as stoppers to limit the distance between the electrodes in the moving and stationary assemblies upon the MEMS motion.

**Figure 9:**
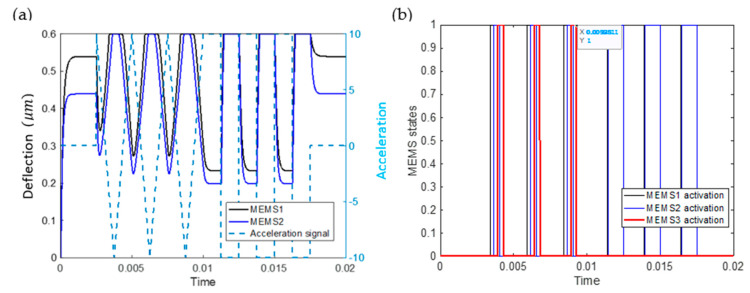
Simulation results for the customized MEMS CTRNN to classify square signal from triangle signal. (**a**) The deflection of the two input MEMS neuron and input acceleration signal. (**b**) the status of the three MEMS neurons, where the status of MEMS3 is considered the network output. The state of the MEMS device is considered to be 1 if pulled-in and 0 otherwise. By the end of each waveform cycle, MEMS3 is correctly on when the input signal is a triangle and is Off when the input signal is a square.

**Figure 10:**
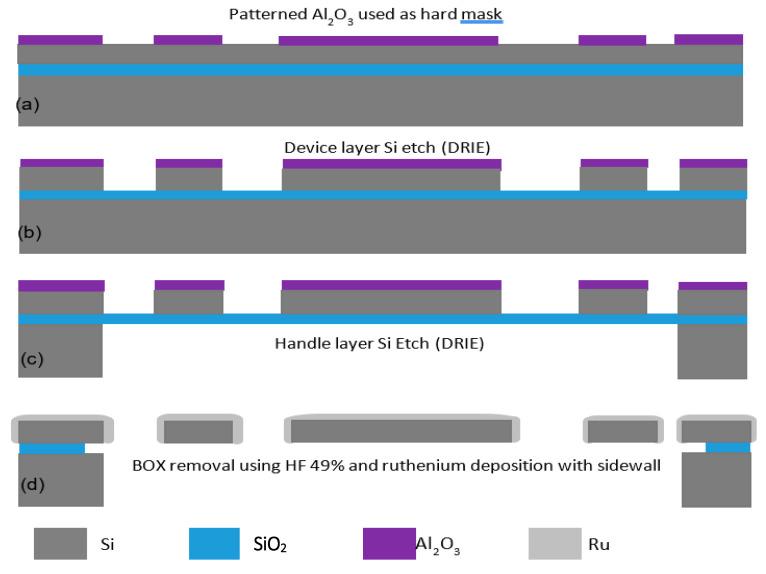
Schematic side view showing the microfabrication process flow: (**a**) Patterned Al2O3 used as hard mask, (**b**) Device layer etch (DRIE) (**c**) Backside etch (DRIE) (**d**) Oxide etch in HF followed by ruthenium deposition with sidewall coverage.

**Figure 11:**
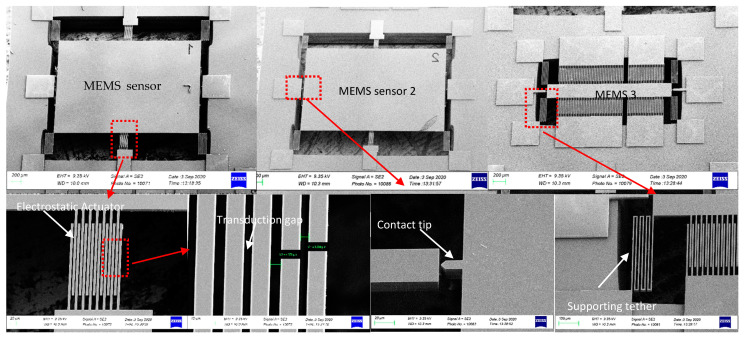
Scanning electron microscope (SEM) views of a fabricated MEMS CTRNN. This novel MEMS network can perform intelligent computing using only bias voltages.

Like the commercial accelerometer network, a new model was developed for the customized network that accounts for electrostatic finger array coupling. Figure 9 shows the high accuracy of the network, using the tuned parameters, to distinguish a square signal from a triangular one. The next step was to fabricate the optimized network. Figure 10 shows the 2-mask micromachining process flow. In this approach, the devices are comprised of a 20–40 µm thick single-crystalline silicon device layer of a Silicon on Insulator (SOI) substrate with a thin coating of Ruthenium. The coating of Ruthenium is for mechanical robustness and low electrical contact resistance at output electrode contacts. The thickness of the substrate’s device layer was chosen to be 30 μm with buried oxide (BOX) layer thickness of 2 μm. First, a 50 nm layer of aluminum oxide (Al2O3) was deposited on the SOI device layer via atomic layer deposition (ALD). The deposited thin film, patterned using the first lithography mask (Figure 10a), is to serve as a hard mask for the following device layer silicon etch. The device silicon skeletons were then carved into the device layer via deep reactive ion etching (DRIE) (Figure 10b). The second mask was used for backside lithography, which was followed by a long DRIE to remove the handle layer underneath the movable parts of the devices (Figure 10c). This is to avoid any potential stiction issues for the large proof masses. The buried oxide layer was wet etched from the backside by a 6-min dip in 49% hydrofluoric acid (HF) solution. The remaining Al2O3 is also removed during this step and the wire bonding pads are partially undercut due to the partial removal of the BOX underneath. Since the ruthenium deposition step is a maskless process, the undercut helps avoid shorts after the metal deposition. Finally, a thin layer of ruthenium (~400 nm thick) was sputtered on the fabricated devices (Figure 10d). The metal coating slightly covers the sidewalls contributing to a high-quality metal to metal electrical contact between the tip of the proof mass and the output electrode. Ruthenium was chosen due to its very high mechanical hardness and excellent wear resistance. A SEM view of a sample of the fabricated MEMS CTRNN is shown in Figure 11.

**Figure 12:**
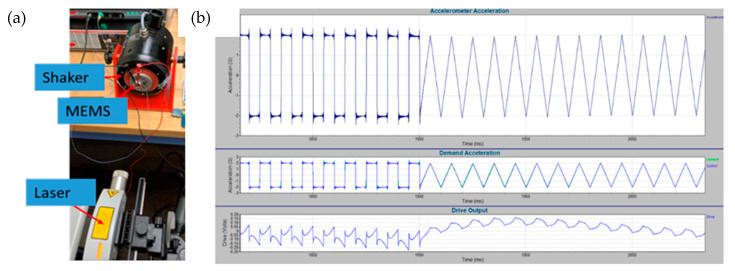
(**a**) The experimental set up to test the MEMS CTRNN, (**b**) samples of the triangle and square acceleration profiles generated by the mechanical shaker.

**Figure 13:**
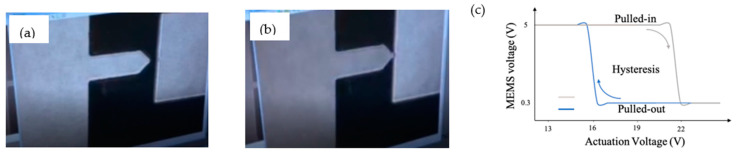
Pull out (**a**) and pull in (**b**) images for MEMS 3. (**c**) A sample hysteresis plot.

A complete experimental set up shown in Figure 12 was designed to test the fabricated MEMS networks. In this setup, the MEMS device is fixed on a shaker. The MEMS response is measured as the difference between the microbeam and substrate base deflections. The shaker is controlled through a dedicated adaptive controller to produce the required signal as shown in Figure 12b. The vibrometer here is used to measure the motion of the entire MEMS structure. However, the actual proof mass deflection cannot be recorded using the vibrometer as the MEMS structure is in-plane. The MEMS response is instead probed electrically at pull-in. The actual MEMS deflection can be found by analyzing images from a digital holographic microscope using edge detection (Figure 13). However, while the computing MEMS3 seems to work as expected as shown in Figure 13, there were issues with MEMS1 & MEMS2 during operation. Specifically, the manual tuning for the design parameters for MEMS1 & MEMS2 devices resulted in having a large proof mass along with very low stiffness tethers for the devices. Thus, they were very vulnerable to shock and vibration, even those happening during handling and mounting the chips. This resulted in multiple supporting tethers breaking. A sample hysteresis plot of MEMS3 is shown in Figure 13c, showing pull-in near 22 V and pull-out near 16 V, providing a wide regime of hysteresis in-between. Here, the MEMS circuit for measuring the output voltage includes a MEMS device, a DC output voltage supply of 5 V and a 200 kΩ resistor. Most voltage drop is across the MEMS device when the device is not pulled in. Once pull-in occurs, the MEMS device acts as an element with low resistance (around 1 kΩ), thus most voltage drop is reported across the external resistor, resulting in the voltage drop across the MEMS device reported in Figure 13c. The reported voltage of 0.3 V at pull-in is a result of the reading being reported using a 1 MΩ input impedance oscilloscope for measurement. Figure correction is attainable by shifting the entire figure by ≈0.3 V.

**Figure 14:**
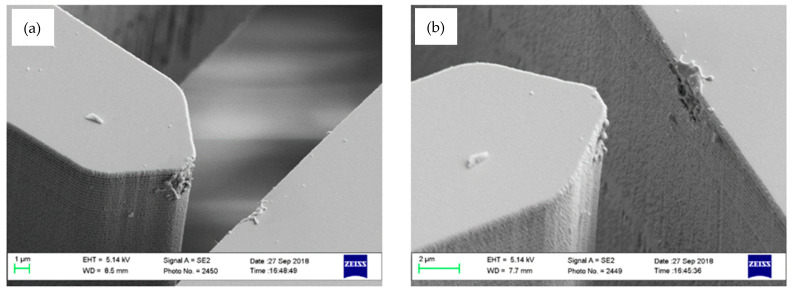
(**a**) SEM view of the contact tip after the long-term operation causing damage to the metal film. (**b**) SEM view of the damaged metal film on output electrode.

The reliability of the MEMS devices based on the number of contacts prior to failure has been characterized. In order to perform the reliability test, the ohmic resistivity between metallic tip of the proof mass and the output electrode (coated with ruthenium thin film) has been monitored for a long-time operation of the device. In this manner, electrostatic actuator of a sample MEMS device was fed by pulse signal with predetermined frequency of 50 Hz and an amplitude that assures pull-in, while the output electrode was biased with a DC voltage of 5 V through a very large resistivity of 100 kΩ. Similar to electrical configuration of the device during the acceleration measurement operation, proof mass was electrically grounded. This operation simulates operating the MEMS device as a switch, which is continuously turned on and off. Contact between the tip of the proof mass and output electrode closes the electrical circuit and results in a DC current through the contact point. In this manner, the ohmic resistivity of metal-metal contact can be simply measured using ohm’s law. An ohmic resistivity of 1.2 kΩ has been measured for the very early cycles of operation. Damage of the thin film metal deposited on the silicon skeleton increases the ohmic resistivity of the contact suddenly at around the 17.5 million cycle mark, which occurred after 4 days of continuous pull-in and pull-off operations. An ohmic resistivity of around 1 MΩ has been measured after the damage of the metal film. Figure 14 shows SEM zoomed-in view of both sides of the contact point after 17.5 million cycles of operation.

## 5. Conclusions

The concept of performing sensing and computing using MEMS devices has great potential for advancing computing in many applications such as wearable devices, however, poses new challenging problems that require new ways of thinking to solve. For example, this novel concept requires optimizing the MEMS design parameters to afford simultaneous sensing and computing. In this paper, however, we adopted a manual training technique that solves intuitively the computing behavior, while ignoring the sensing limitation. While our simulation shows a great response, the real implementation and fabrication of this MEMS CTRNN network revealed that its sensing mechanical parameters (i.e., mass and stiffness) while accommodating the required computing aspect, were too sensitive to shocks resulting in their mechanical failure. Our ongoing approach involves using common machine learning techniques such as genetic algorithm and Back Propagation Through Time (BPTT) among other methods to optimize the MEMS parameters to satisfy both the computing and sensing requirements of the MEMS CTRNN network. We also plan to investigate the capability of MEMS CTRNN in more complex classification applications with relatively long-term time-series patterns such as those that occur in motion sensor data involving human activities.

## Figures and Tables

**Table 1:** MEMS parameters.

MEMS Parameter	Value
Length *(l)*	9 mm
Width *(b)*	5.32 mm
ε	8.85 × 10^−12^ F/m
Gap *(d)*	42 μm
Stiffness *(k)*	215 N/m
Mass (m*)*	143 mg
Dampign cofficient (c)	0.351 N. s/m
Bias MEMS1 ($V_{b,1}$)	50 V
Bias MEMS2 $\left(V_{b,2}\right)$	50 V
Bias MEMS3 ($V_{b,3})$	50 V
Weight MEMS3→ 1 ($w_{31}$)	1.5
Weight MEMS3 → 2 $w_{32}$	−1
Threshold deflection ($x_{s})$	30 μm

## Appendix A

Schematics and dimensions of the computing MEMS, MEMS3, fabricated in Section 4.

## Appendix B

Schematics and dimensions of the input-layer MEMS devices: MEMS1 and MEMS2, fabricated in Section 4. The two MEMS devices are shown below, respectively.
